# A Review of High-Sensitivity SERS-Active Photonic Crystal Fiber Sensors for Chemical and Biological Detection

**DOI:** 10.3390/s25226982

**Published:** 2025-11-15

**Authors:** Jiaying Luo, Jia Sun, Huacai Chen, Chunliu Zhao, Manping Ye

**Affiliations:** College of Optical and Electronic Technology, China Jiliang University, Hangzhou 310018, China

**Keywords:** Surface-Enhanced Raman Scattering (SERS), Photonic Crystal Fibers (PCFs), sensors, chemical sensors

## Abstract

This review critically surveys the emerging integration of Surface-Enhanced Raman Scattering (SERS) with photonic-crystal fibers (PCFs) for chemical and biological detection, an area still scarce in the literature. SERS exploits electromagnetic and chemical enhancement to overcome the intrinsic weakness of Raman scattering, while PCF offers low transmission loss and a strong evanescent field that further amplify the signal. The structural designs of PCF, encompassing solid-core and hollow-core variants, are discussed and their respective advantages in different sensing scenarios are presented. Applications in chemical detection, biomedicine, and explosive identification are detailed, demonstrating the versatility and potential of PCF-SERS sensors. Future efforts will focus on robust PCF geometries that guarantee stable and reproducible signals, AI-driven spectral algorithms, hybrid fibre architectures and scalable manufacturing. These advances are expected to translate PCF-SERS from bench-top demonstrations to routine deployment in environmental monitoring, clinical diagnostics and food-safety control.

## 1. Introduction

Raman spectroscopy is an optical spectroscopy method based on spontaneous or excited Raman scattering, which obtains information on molecular vibration and rotation by analyzing the scattering spectra of molecules interacting with incident light [1]. Despite its unique ability to identify molecular fingerprints and the advantages of non-destructive detection, the application of Raman spectroscopy is limited by its low signal intensity. Raman scattering is intrinsically weak: as only a small fraction of the incident light is inelastically scattered, and this scattering propagates in all directions. This limitation is particularly noticeable in biological and chemical analyses, where low concentration samples or molecules with weak Raman scattering cross sections are difficult to detect efficiently [2,3]. To this end, surface-enhanced Raman spectroscopy (SERS) emerged-its core relies on Localized Surface Plasmon Resonance (LSPR)-mediated electromagnetic field enhancement [4], which greatly improves Raman detection sensitivity (even enabling single-molecule detection) [5].

The core advantage of SERS technology is its ability to achieve significant enhancement of Raman scattering signals on the surface of metal nanostructures, often by a factor of a million or more. This enhancement not only overcomes the limitation of weak signals in conventional Raman spectroscopy, but also preserves the molecular ‘fingerprint’ information provided by Raman spectroscopy, which enables SERS to have a wide range of potential applications in the fields of chemistry, materials science and life science [6,7,8,9]. For example, in environmental monitoring, SERS can be used to detect trace pollutants [10,11,12,13]; in the biomedical field, SERS can be used for highly sensitive detection of biomolecules [14,15,16,17]; and in the food safety field, SERS can be used for the detection of various toxins [18,19,20]. Rough precious-metal surfaces, especially nanoscale gold and silver particles, give the strongest enhancement and are the most widely used substrates [21].

Yet conventional SERS remains limited. It struggles with biological samples and small volumes in label-free mode. Fluorescent or other interfering signals are hard to remove. Real-time remote sensing is also difficult. [22,23]. In addition, conventional SERS sensors may potentially face challenges in optimizing light delivery and collection, affecting sensitivity, signal stability and signal-to-noise ratio (SNR). All these limitations emphasize the need for more innovative and adaptable sensing solutions using different SERS substrates [24].

In this context photonic-crystal fibres (PCFs) offer a new SERS substrate. PCF behaves much like conventional plasmonic nanomaterials: both sculpt the optical field—NPs via LSPR, PCF via evanescent or band-gap confinement; both are spectrally tunable—NPs by size or shape, PCF by core/cladding design; and both maximize hot spots—NPs in the gaps between particles, PCF by aligning those gaps with the guided mode [25]. The PCF sensors, when combined with SERS, exhibit distinct advantages compared to conventional SERS techniques (see Table 1). These advantages include a significantly reduced Raman background signal, an expanded active sensing volume, high spectral resolution, and independence from water absorption. They solve long standing problems by improving light analyte plasmon coupling instead of relying on plasmonic properties. The porous core compresses guided light and extends its contact with Au/Ag NPs and analytes along the fibre. As a result, these innovative PCF-SERS sensors have garnered considerable attention from the scientific community.

**Figure 1:**
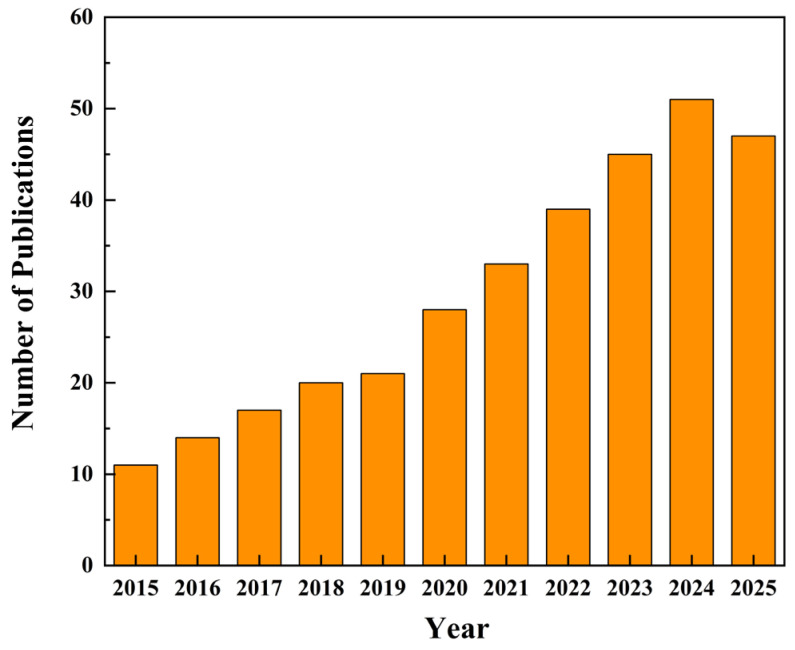
Annual publication volume trend chart.

**Figure 2:**
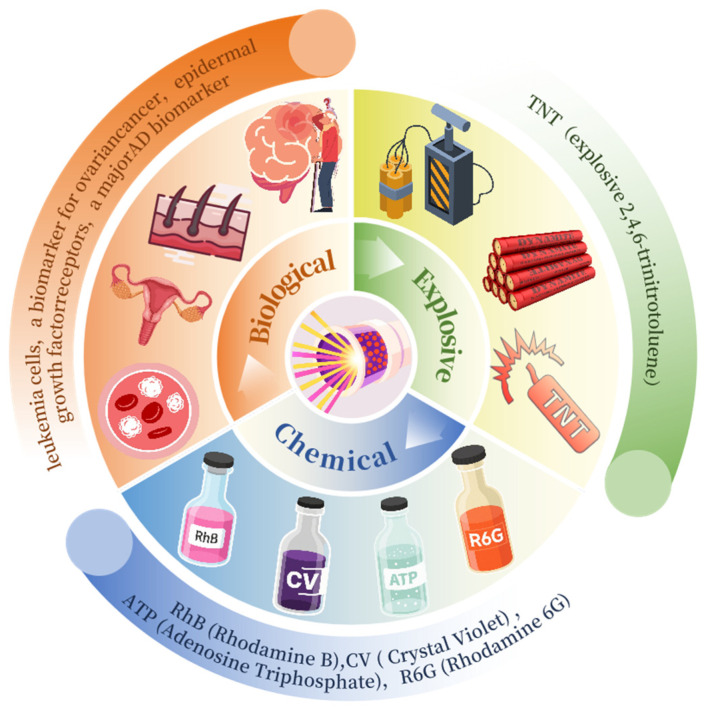
Schematic diagram of an application example based on PCF-SERS sensors.

This review critically examines the emerging convergence of SERS and PCF for chemical and biological sensing, systematically evaluating current applications and charting future research trajectories. Figure 1 displays the annual number of PCF–SERS publications from 2015 to October 2025; the tally exceeded 50 in 2024 and continues to climb, underscoring the urgent need for a comprehensive overview of progress and challenges For the convenience of researchers, these technologies are specifically subdivided into three key application areas so that researchers can quickly locate the content of interest according to their own professional background (see Figure 2). Each section lists the relevant research results in detail in the form of a table, which facilitates the visual comparison of the perceived performance of each technology and deepens the understanding. The review is clearly structured: Section 2 briefly introduces the basic principles of SERS, including electromagnetic enhancement and chemical enhancement. In Section 3, we demonstrate a series of promising PCF-based sensor designs, including hollow-core PCF, solid-core PCF, followed by Section 4, which focuses on the analysis of PCF-SERS sensors in several key areas, such as chemicals, biomedicine, and explosives. Section 5 discusses future trends of SERS technology and points out the current challenges and opportunities, which point out the direction for research in this field. By integrating the high sensitivity of SERS with the unique optical properties of PCF, this review demonstrates the tremendous potential of this technology in fields such as chemical analysis, biomedical detection, and explosive identification. It is anticipated that this work will offer valuable guidance and reference for future analytical investigations employing this advanced spectroscopic technique.

## 2. SERS Overview

**Figure 3:**
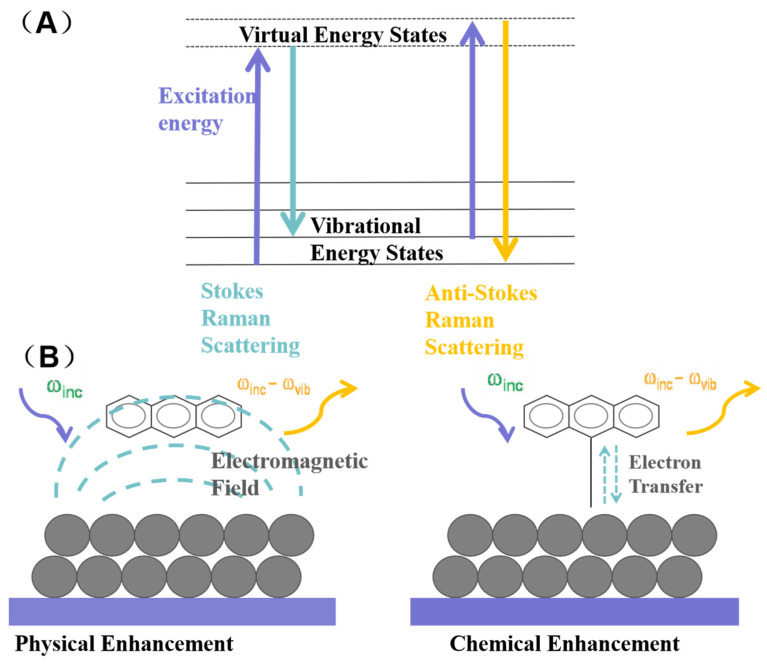
(**A**) The energy level diagram for Raman scattering. (**B**) Schematic illustration of electromagnetic (**left**) and chemical enhancement (**right**) of Raman scattering signals of a molecule adsorbed on the surface of noble metal nanoparticles.

The Raman spectrum, a form of vibrational spectroscopy, occurs when incident light strikes a sample, causing inelastic collisions between photons and material molecules. This energy exchange alters the photons’ frequency, a phenomenon known as Raman scattering [1,26]. In Raman scattering, the energy of a photon can either increase or decrease, leading to two types of scattering: Stokes and anti-Stokes (see Figure 3A). Stokes scattering happens when a photon interacts with a material, exciting the material to a higher-energy virtual state and then relaxing to a higher-energy vibrational state, resulting in a lower energy photon. Conversely, anti-Stokes scattering occurs when a molecule in a higher-energy vibrational state is excited to a virtual state by a photon and then relaxes to a lower vibrational energy level producing a higher-energy photon.

The phenomenon of SERS was first observed in 1974 when researchers found that pyridine molecules adsorbed on a rough silver electrode exhibited significantly enhanced Raman signals. SERS leverages nanoscale roughened metal surfaces to enhance signal intensity, allowing for the detection of trace amounts of molecules [4,27]. SERS is an advanced Raman spectroscopy technique that leverages nanoscale roughened metal surfaces to significantly enhance signal intensity. This enhancement allows for the detection of trace amounts of molecules, and in some cases, even individual molecules. As a result, SERS stands out as one of the few analytical techniques capable of achieving such an extraordinary level of sensitivity [28,29,30]. The exact enhancement mechanism of SERS is still not fully understood, but it is widely accepted that two main mechanisms contribute to the enhancement: the physical enhancement mechanism and the chemical enhancement mechanism [31]. The physical mechanism involves the generation of a strong localized electromagnetic field when incident light resonates with the localized surface plasmon resonance (LSPR) frequency of the SERS substrate. This field enhances the Raman polarization of the analyte molecules and is considered the dominant contributor to SERS (see Figure 3B, left). The chemical mechanism involves charge transfer between the metal surface and the adsorbed molecules, altering the electron density distribution on the molecular surface and leading to a significant enhancement of the Raman signal (see Figure 3B, right) [18,32].

Despite its high sensitivity of SERS technology makes it an ideal detection method, but its application is limited by signal stability and reproducibility. The introduction of PCF offers new possibilities for addressing these challenges. PCF’s unique optical structure, particularly its micro-porous design, allows for the incorporation of metal nanoparticles such as gold and silver into the fiber’s air channels. This integration increases the number of “hot spots”, regions where the localized electromagnetic field is significantly amplified due to the proximity of nanoparticles, thereby enhancing the Raman signals of nearby molecules. Additionally, PCF’s light field confinement property ensures a longer interaction path between the light and the analyte molecules, leading to more efficient energy transfer and a stronger Raman scattering signal. The low transmission loss of PCF also contributes to minimal signal attenuation. These structural advantages make PCF an excellent substrate for achieving high-sensitivity and high-selectivity detection in SERS applications, and Gao et al. [33] further elaborated on PCF’s role in optimizing SERS performance: optofluidic integration with PCF enhances light-analyte interaction and enables precise control of sample volume (down to nL level)—a feature that further confirms such structural advantages of PCF.

In PCF-SERS platforms, signal amplification is governed almost exclusively by electromagnetic enhancement: the solid core’s intense evanescent field drives strong LSPR of surface-bound Au/Ag NPs, whereas the hollow core extends the light–NP–analyte dwell time, multiplying hot-spot events [33]. Chemical enhancement remains minor because low-affinity Au/Ag NPs are deliberately chosen to suppress metal–analyte charge transfer.

## 3. Types and Principles of PCF-SERS Sensors

Initially, SERS substrates were typically roughened gold and silver electrodes. These rough metal surfaces feature numerous “hot spots” and provide a high specific surface area, which is beneficial for signal enhancement. However, the positions of these “hot spots” are not adjustable, making it challenging to conduct in-depth studies on the surface-enhanced Raman mechanism. In response to this challenge, the swift progress in nanomaterials in recent years has spurred the integration of photonic crystals into the design of SERS sensors. Photonic crystals have emerged as a popular choice for SERS substrate preparation due to their unique optical properties and tunability [34,35,36]. PCF, which are two-dimensional photonic crystals, are also referred to as porous fibers or micro structured fibers. Their cladding region is characterized by a periodic arrangement of micro-air holes that run parallel to the fiber’s axial direction. Owing to their numerous advantages, including low cost, ease of coupling with detectors, and the integration of sample preparation and detection, PCF has garnered significant attention from researchers for the development of detection substrates [34].

### 3.1. Solid-Core Photonic Crystal Fibers (SC-PCF)

**Figure 4:**
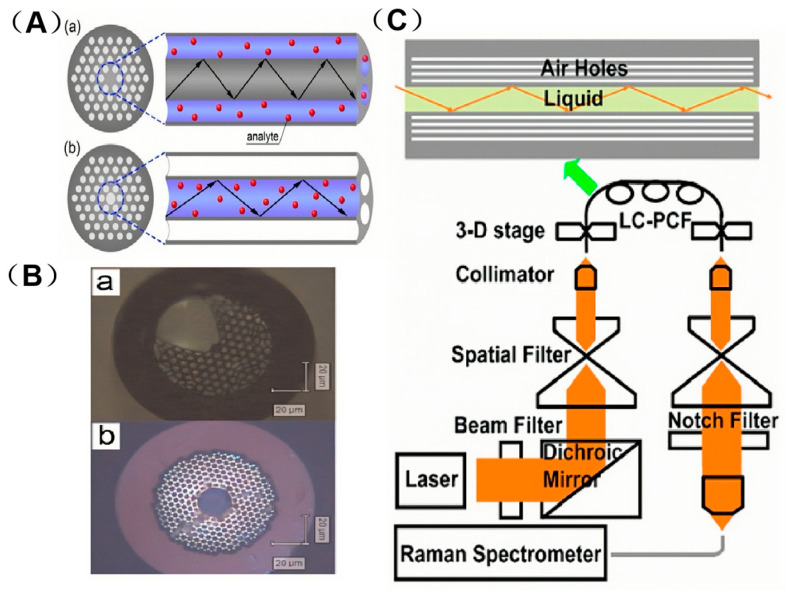
(**A**) Schematic example of PCF with solid (**a**) and hollow cores (**b**). Adapted with permission from ref. [39]. (**B**) Microscope image of a cross section of (**a**) SC-PCF and (**b**) HC-PCF. Adapted with permission from ref. [40]. (**C**) Schematic illustrations of light guiding in the liquid core by total internal reflectance in a LC PCF top and a fiber Raman setup. Adapted with permission from ref. [44].

PCF can be categorized into two main types based on their light-conducting mechanisms. One type is Solid-core Photonic Crystal Fibers (SC-PCFs), also known as refractive-index-guiding PCF. The structural schematic and cross-sectional microscopic images of these fibers are illustrated in Figure 4A(a),B(a). In SC-PCF, the core region is composed of a solid quartz material, while the cladding features an array of micro-air holes. This design creates a total internal reflection (TIR) light-guiding mechanism, where the effective refractive index of the cladding, due to the presence of the air holes, is lower than that of the core region. As a result, light is confined within the core through TIR. For detection purposes, the refractive index of the solution being analyzed must be lower than that of the core material. The cladding’s micro-air hole structure ensures that its effective refractive index remains smaller than that of the core, thereby maintaining efficient light guidance through total internal reflection [37,38,39,40]. For detection, the solution to be analyzed must be mixed with the metal nanoparticle sol and then injected into the porous channel of the SC-PCF. Meanwhile, the light source propagates within the solid core layer of the fiber [34]. However, the application of SC-PCF based biosensors often necessitates precise alignment under a microscope. This requirement may compromise the reliability of SERS measurements and could pose limitations to their practical end-use applications. This evanescent field enhancement (concentrating light at the core surface) aligns with metal NPs’ light-modulating ability: both boost SERS excitation, NPs through LSPR hot-spots and SC-PCF through an intensified evanescent bath that bathes adjacent Au/Ag NPs. In this context, Beffara’s team has introduced a range of innovative Suspended-core Photonic Crystal Fibers (SuC-PCF)in which the suspended silicon rods are replaced by thin silicon rings, this innovative design modification not only allows for an order of magnitude increase in the surface area available for attaching nanoparticles [41], but also facilitates the development of tapered suspended-core photonic crystal fibers (tapered-SuC-PCF). These tapered fibers are designed to enhance coupling efficiency and improve the reliability of measurements [42]. SC-PCF, with its low loss and high evanescent field power, is particularly suitable for chemical sensing that requires high sensitivity. Beffara’s SuC-PCF structural optimization is supported by Issatayeva et al. [43]. Their simulations and biomolecular tests showed 3 μm-core (5 cm). SuC-PCF has higher Raman intensity than 2 μm-core versions at typical nanoparticle densities—confirming core size/length enhance its biomolecular sensing suitability. This core size tuning reflects metal NPs’ adaptability: just as 50 nm AgNPs suit visible-light SERS, 3 μm-core SuC-PCF is tailored for biomolecular sensing, matching structure to analytes. SiC-PCF’s side channel enables uniform AuNP loading.

### 3.2. Hollow-Core Photonic Crystal Fibers (HC-PCF)

The other type is Hollow-core Photonic Crystal Fibers (HC-PCFs), also known as photonic bandgap-guiding PCF. Structural schematic and cross-sectional micrographs appear in Figure 4A(b),B(b). In HC-PCF, the core region of the fiber is a large air pore, while the cladding consists of micro-air holes arranged in a periodic, cyclic pattern, forming a two-dimensional photonic crystal structure. The bandgap effect of these photonic crystals prohibits light within a specific frequency range and propagation constant from propagating through the cladding, instead confining it to the air holes in the core region [37,39,40]. This band-gap confinement parallels metal NPs’ LSPR: both trap light to enhance interactions—NPs for local field amplification, HC-PCF for prolonged light–Au/Ag NP/analyte contact. When the core is filled with liquid the fibre is often called Liquid-core Photonic Crystal Fiber (LC-PCF), ensuring the metal-NP hot spots fully overlap the guided mode. For detection the mixed solution is injected into the hollow core where light propagates; total internal reflection at the fluid–glass interface produces multiple reflections [34]. PCF-SERS sensors leverage this confinement property to enable continuous interactions between the excitation light source and the analyte molecules, or between the analyte molecules and the discrete exciton nanoparticle aggregates. These interactions, in turn, generate a strong Raman spectrum [39] (see Figure 4C). It is evident that once the excitation light source is introduced into the optical fiber, the Raman-scattered light generated also continues to propagate along the fiber and ultimately reaches the detector [44]. Thus HC-PCF, confining light via the photonic bandgap effect, delivers excellent performance in liquid-sample SERS detection.

### 3.3. Comparison of SERS Signal Distribution Between SC-PCF and HC-PCF

**Figure 5:**
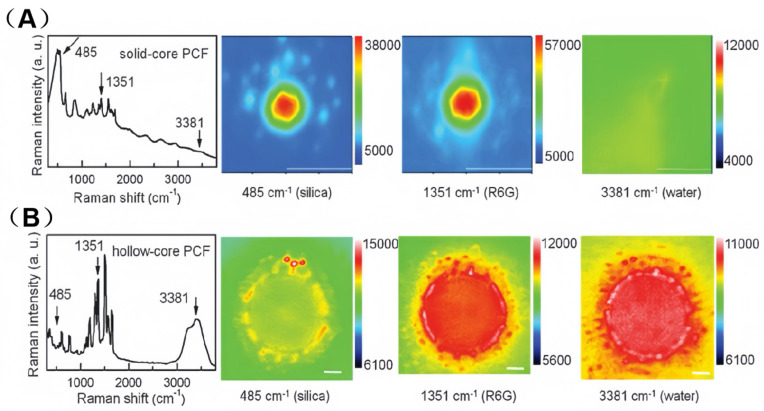
SERS spectra of 10^−5^ M R6G and hyperspectral Raman images of silica, R6G, and water from SC-PCF (**A**) and HC-PCF (**B**) with immobilized Ag nanoparticles of ~0.5 particle μm^−2^ in coverage density. Adapted with permission from ref. [39].

To explicitly illustrate how the distinct light-guiding mechanisms of SC-PCF and HC-PCF differentially impact their SERS signal distributions and analyte interactions, Figure 5A,B present comparative spectral and hyperspectral Raman imaging results for the two configurations.

In the SC-PCF (as shown in Figure 5A), optical transmission primarily relies on the core mode. This mode significantly contributes to both the Raman signal of silica (via direct excitation) and the Raman signal of rhodamine 6 G (R6G) (via rapid field interaction). As a result, the SERS signal of R6G is effectively confined to the core region. The hyperspectral imaging results of the cladding region (middle two images of Figure 5A) reveal that the junction of the three air channels (each resembling a silicon optical waveguide) also contributes weakly to the signal. However, the Raman signal of water could not be detected in the SC-PCF [39].

In contrast, Raman imaging of the air-core PCF revealed a distinctive dashed ring pattern of silica Raman intensity distribution (left image in Figure 5B). This phenomenon clearly indicates that the triple-junction structure between the fiber core and the two adjacent air channels enables light guidance, specifically the forward propagation of the silica signal. Additionally, the Raman signal distributions of R6G and water (right two images in Figure 5B) are confined within the liquid-filled fiber core. This suggests that the liquid core mode plays a dominant role in the measured Raman intensity [39].

The distinct features of SC-PCF and HC-PCF give them unique advantages in different application scenarios. By making appropriate selections and optimizations, the performance of SERS sensors can be further enhanced. PCF-SERS sensors can be readily integrated with microfluidic channels, making them highly suitable for the detection of small biological molecules [45], molecules in human body fluids [46,47] and even explosives [48] and highly toxic substances [44].

## 4. Application of PCF-SERS Sensors

The development of PCF-SERS probes suitable for ultra-sensitive and in situ detection is a topic of significant scientific interest. Two important optical designs have been explored. In the original design, the optical fiber serves as a single waveguide for both light transmission and collection of Raman scattering signals from various SERS-active substrates. This particular configuration is commonly referred to as an external fiber-optic SERS sensor [49]. On the other hand, the second approach, known as intrinsic fiber-optic SERS sensors, involves integrating metallic SERS-active nanostructures directly with the optical fiber, typically at its tip. In this design, the fiber-optic probe functions both as a waveguide for light transmission and as a platform for SERS activation [50,51]. This integration not only enhances the interaction between light and matter but also significantly improves the sensitivity and spatial resolution of the sensor, thereby offering new possibilities for ultrasensitive detection and in situ analysis. While PCF is already a powerful type of optical fiber, PCF-SERS sensors provide exceptional versatility and have had a transformative impact on several key applications.

In the realm of chemical detection, the sensor’s high precision and accuracy enable it to acutely capture and accurately profile trace amounts of chemicals. In biomedical testing, PCF-SERS sensors serve as a powerful tool for identifying and quantifying biomolecules. They have revolutionized traditional medical diagnostic techniques, providing a new impetus for early screening and accurate disease diagnosis, and advancing biomedical research. In the detection of special microorganisms and explosives, the high sensitivity of SERS technology allows PCF-SERS sensors to accurately detect their traces at very low concentrations. This capability provides crucial support for public safety protection. The excellent adaptability of PCF-SERS sensors in diverse and complex environments underscores their importance in advancing scientific research. Specific applications are detailed in the following subsections.

### 4.1. Detection of Chemical Substances

**Figure 6:**
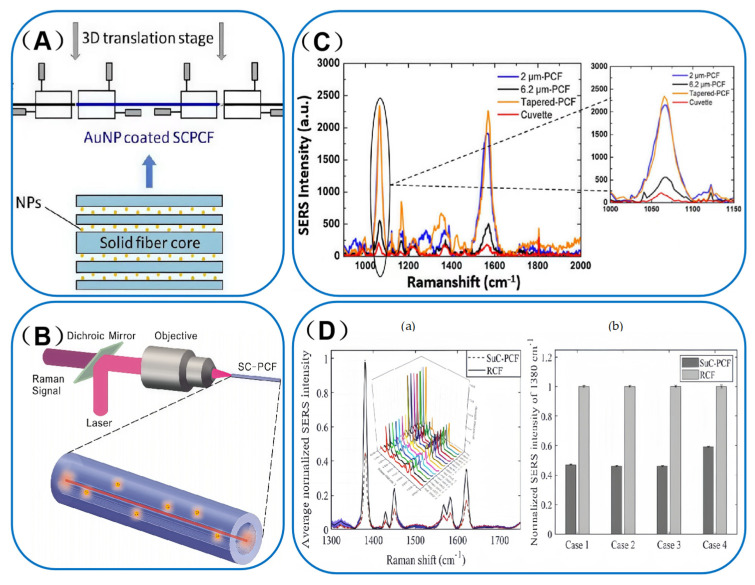
(**A**) Experimental setup for transmission measurement of the probe. Adapted with permission from ref. [45]. (**B**) Schematic diagram of the setup for backscattered SERS measurements (sizes are not to scale). Adapted with permission from ref. [56]. (**C**) SERS spectra of ATP molecules measured with cuvette-1 mm, 2 µm-PCF, 6.2 µm-PCF and the tapered-PCF, and zoomed image of the spectral intensity of 1080 cm^−1^ peak. Adapted with permission from ref. [42]. (**D**) (**a**) Average SERS spectra obtained with a single SuC-PCF and RCF over seven measurements. (**b**) Normalized RI of 1380 cm^−1^ peak of 2-NT obtained with RCFs and SuC-PCF sensors. Adapted with permission from ref. [41].

The PCF-SERS sensors have become a highly promising technology for the real-time, in situ detection of a wide variety of chemical analytes. Figure 6 compiles the sensor architectures and experimental setup with SERS spectra for all surveyed chemical analytes. Their unique capabilities and high sensitivity have established them as an indispensable tool in the field of chemical research [52].

In the field of chemical substance detection, PCF-SERS sensing technology has yielded significant results. For instance, researchers employed SC-PCF and assembled gold nanoparticles (AuNPs) on the inner walls of the cladding pores through multilayer deposition. They adopted an offset emission method, utilizing a self-built device (as shown in Figure 6A) for UV-visible characterization of the resulting SERS substrate. Thanks to the increased overlap of excitation modes with AuNPs, the detection limit for the rhodamine B (RhB) solution was successfully reduced to 10^−7^ M, providing an important reference for chemical sensing applications [53].

Building on these findings, researchers have increasingly turned their attention to HC-PCF. In recent years, a research team has focused on HC-PCF and designed both liquid-filled HC-PCF probes and LC-PCF probes, using silver nanoparticles (SNPs) as SERS substrates. Liquid-filled HC-PCF probes provide a 100-fold increase in sensitivity compared to direct sampling at relatively high concentrations. However, at low concentrations, detection is hindered due to the background enhancement from SNPs. In contrast, LC-PCF probes enable the detection of R6G at concentrations as low as 10^−10^ M. This enhanced sensitivity is attributed to the liquid within the HC-PCF, which improves light confinement and increases the number of particles involved in the SERS process [54].

The Maung team [55] conducted numerical simulations and hyperspectral Raman imaging of refractive index-guided SC-PCF featuring three distinct air-cladding microstructures. Among these, the SuC-PCF exhibited superior performance, enabling the detection of 1 × 10^−10^ M R6G within a sampling volume of 7.3 μL of aqueous solution. These findings underscore the potential of SC-PCF as powerful platforms for chemical research.

Figure 6B schematically illustrates a solid-core side-channel photonic crystal fiber equipped with a backscattering SERS measurement device. Its unique triangular cladding structure removes one-third of the cladding to form a cavity, facilitating rapid liquid penetration and expanding the interaction area. This design features low transmission loss, high evanescent power, and single-mode propagation characteristics at the excitation wavelength of 632.8 nm. When tested with a mixture of gold nanoparticles (AuNPs) and R6G solution, the detection limit is as low as 50 fM. The SERS signal intensity exhibits a cumulative effect with increasing fiber length, as analyzed by a numerical analytical model. Sensitivity can be enhanced by increasing the fiber length [56].

With the continuous advancement in optical fiber design, Beffara’s team has effectively enhanced SERS detection performance by optimizing the fiber structure, such as developing tapered and ring-core configurations. In terms of detection outcomes, these optimized fibers achieved high sensitivity for target molecules (e.g., ATP and 2-NT) with excellent reliability and reproducibility. They have particularly overcome the limitations of traditional planar or colloidal nanoparticle SERS platforms by increasing the light-analysate interaction area and improving coupling efficiency.

Firstly, the team calculated the overlap integral of the electromagnetic field distribution between SuC-PCF and the laser beam using the finite element method and simulated the deviation. They then fabricated SuC-PCF and tapered SuC-PCF with different core diameters via the stacking-drawing process and functionalized them. As shown in Figure 6C, the results indicated that the SuC-PCF with a 6.2 μm core diameter exhibited the highest coupling coefficient (approximately 63%). The tapered SuC-PCF showed a coupling efficiency about 60% better than that of the 2 μm PCF (around 21%) and demonstrated good repeatability. In SERS measurements, the tapered SuC-PCF performed exceptionally well with a 1 mM ATP solution, achieving sensitivity about four times higher than that of the 6.2 μm PCF. It also exhibited similar and slightly higher peak sensitivity compared to the 2 μm PCF, which was 12 times better than that of a standard cuvette. Additionally, the tapered SuC-PCF outperformed the 2 μm PCF in reliability tests. The “plug and play” system tested well with both 1 mM and 100 nM ATP solutions [42].

Secondly, the team also simulated ring-core fibers (RCFs) and SuC-PCF using the finite element method. Subsequently, RCFs were fabricated via the stacking-drawing process, with optimization of the preparation conditions. After functionalization, the fibers were injected into a 2-nitrotoluene (2-NT) solution for detection. As shown in Figure 6D, the signal intensity of the RCF in the 2-NT solution was twice as high as that of the SuC-PCF, with excellent reproducibility [41].

In summary, while PCF-SERS spectroscopy holds remarkable advantages and wide applications, the preparation of SERS enhancement substrates remains a critical factor. Traditional planar SERS substrates often require pre-concentration for detection. In contrast, PCFs enable sensitive, real-time, in situ detection of liquid-phase molecules but may involve complex preparation processes. This study centers on capillaries and SuC-PCF. Initially, a capillary-based SERS trace detection method is proposed. By pre-mixing Au NPs with the analyte, optimizing NP size, and using capillary grazing incidence and multimode interference, rapid CV trace detection in water is achieved. This method boasts a detection limit of 10^−9^ M and an enhancement factor of 108. Secondly, to address weak light field interaction in probes, an anti-coupling mechanism in SuC-PCF is introduced. An efficient substrate preparation controls Ag NPs deposition. The resulting SuC-PCF is ultra-sensitive, with a CV detection limit of 10^−12^ M [57]. This study focuses on key issues related to SERS substrates and innovatively employs two types of microstructured fibers. It presents effective strategies to solve the problem of weak laser intensity in probes, thereby enhancing the sensitivity and practicality of SERS technology. Reusable, highly sensitive fiber SERS probes have been successfully fabricated and applied in water monitoring and pesticide residue detection, demonstrating significant theoretical and practical value. However, the study acknowledges that it may not fully explore interference factors and countermeasures for complex real samples, indicating areas for future improvement.

The Analytical Enhancement Factor (AEF) for chemical analytes in PCF-SERS sensors. Planar Au/Ag films or colloids stop at 10^6^–10^8^, barely adequate for ultra-trace work. PCF-SERS improves the enhancement factor by 1–3 orders of magnitude: the evanescent field of SC-PCF and the band-gap confinement of HC-PCF compress the guided light into every plasmonic hot spot far more efficiently than conventional planar substrates.

The Limit of detection (LOD) for chemical analytes in PCF-SERS sensors is fixed by the synergy between fibre geometry and plasmonic nanomaterials. For liquid samples AgNP-functionalised HC-PCF matches SC-PCF LODs. The hollow core holds the analyte directly, eliminating diffusion loss into cladding pores and maximising light–analyte interaction time. However, the band-gap position drifts with refractive index, so complex organics still fare better with SC-PCF.

When chemical stability is paramount SC-PCF paired with AuNPs offers a practical compromise. AuNPs provide weaker LSPR enhancement and higher dye LODs, yet their superior oxidation resistance ensures reliable operation in acidic or basic matrices where AgNPs degrade. Table 2 summarizes the reported PCF-SERS-based chemical substances.

### 4.2. Detection of Biomedicine

#### 4.2.1. Disease Biomarkers

In biomedicine, the high selectivity of PCF-SERS sensors offers new possibilities for detecting disease biomarkers. As shown in Table 3 Applications of PCF-SERS sensors in biomedicine, these sensors have exhibited effective performance in detecting various disease biomarkers across multiple biomedical scenarios, providing valuable references for clinical applications. Disease biomarkers play a crucial role in disease diagnosis, encompassing a diverse array of types, including proteins, nucleic acids, and small-molecule metabolites [58,59,60]. Among them, proteins, as a widely used class of disease markers, have demonstrated a variety of important clinical applications at all stages of disease from early to late stages. These values include, but are not limited to, early diagnosis of disease, real-time monitoring of the therapeutic process, and accurate determination of post-treatment relapse [61,62,63]. However, protein disease markers are often present at low concentrations in clinical samples and are susceptible to interference from high-abundance components. Achieving highly specific and sensitive detection of these markers has long been a significant challenge in clinical diagnostics. Currently, several methods are available for the detection of disease markers, including colorimetric assays [64], electrochemiluminescence [65], biomass spectrometry [66], and immunoassay [67]. However, these methods still face challenges when confronted with low concentrations of biomarkers. In recent years, the integration of SERS technology with PCF has emerged as a novel solution to address this challenge.

**Figure 7:**
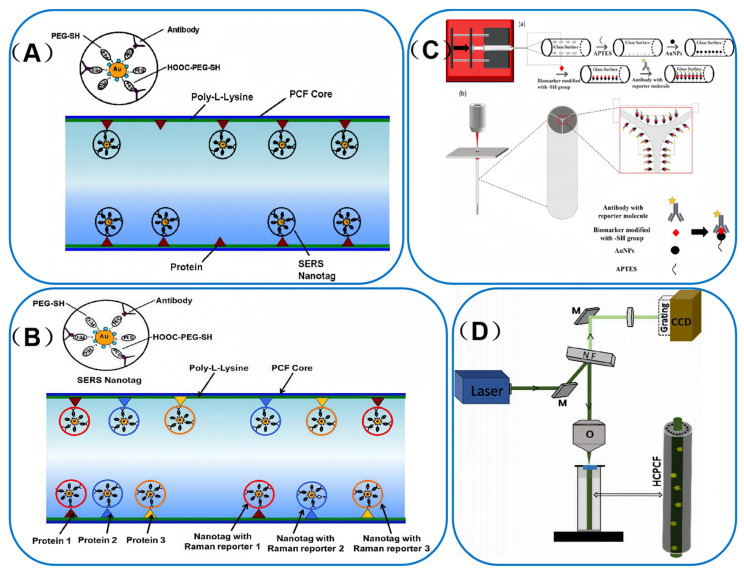
(**A**) Schematic representation of the binding of anti-EGFR antibody conjugated SERS nanotag to the cancer protein (positive control, from A431 cells) immobilized on the inner wall of the core of HC-PCF. Adapted with permission from ref. [68]. (**B**) schematic of the binding of SERS nanotags to immobilized biomarkers inside the core of HC-PCF for multiplex detection. Adapted with permission from ref. [69]. (**C**) (**a**) Schematic of the functionalization process inside the holes of the PCF for biomarker sensing. Fiber is connected to a syringe needle. (**b**) Coupling of PCF to objective lens of Raman spectrometer and backscattering configuration of the fiber for signal collection. Zoom in on the fiber end face and holes with the attached protein and read out Raman tag. Adapted with permission from ref. [70]. (**D**) Schematic of the fiber-enhanced Raman spectroscopic setup. (M, mirror; NF, notch filter; and O, microscopic objective). Adapted with permission from ref. [71].

In 2012, a PCF-SERS sensing platform [68] was reported for ultrasensitive detection of cancer proteins in ultra-low sample volumes. In their study, three different SERS nanotags were used for the first time to detect epidermal growth factor receptor (EGFR) biomarkers in oral squamous carcinoma cell lysates. The team pre-coated the inner wall of the fiber with Poly-L-lysine (PLL) to create a highly efficient “pocket” structure that specifically captures and immobilizes the target protein. Subsequently, the immobilized proteins were detected using antibody-coupled SERS nanolabels, as illustrated in Figure 7A. In this study, the system achieved a minimum detection concentration of 100 pg/mL, which was a significant milestone in the field at the time.

Building on this progress, in 2014, Dinish et al. published a groundbreaking study that combined SERS technology with HC-PCF to achieve the simultaneous detection of multiple cancer biomarkers. This innovative approach leveraged the unique advantages of SERS and HC-PCF to enhance detection sensitivity and multiplexing capabilities [69]. Three distinct SERS nanoprobes were developed using different Raman reporter molecules: cyanine 5 (Cy5), malachite green isothiocyanate (MGITC), and naphthalene thiol (NT). They were synthesized by functionalizing SERS nanoparticles with poly-L-lysine (PLL) to ensure stable and specific binding. The design of this biosensing system is illustrated in Figure 7B. The biosensing platform successfully demonstrated the simultaneous detection of epidermal growth factor receptor (EGFR) markers in oral squamous cell carcinoma, as well as alpha-fetoprotein (AFP) and alpha-1-antitrypsin (A1AT) markers in Hep3b cells. This achievement not only represents a significant advancement in the detection of low-concentration biomarkers but also holds great promise for early disease diagnosis and personalized medicine. The selected reporter molecules were chosen for their unique Raman spectral signatures, which enabled the simultaneous detection of multiple targets without interference. To further enhance the specificity of the biosensing platform, these SERS-activated nanoparticles were conjugated with antibodies targeting specific cancer biomarkers. This strategy allowed for the efficient and accurate detection of multiple biomarkers in complex biological.

In a similar vein, Beffara et al. [70] developed highly repeatable and reproducible SERS-activated SuC-PCF probes. Figure 7C(a) illustrates a detailed schematic of the functionalization process within the PCF pore for biomarker sensing, while Figure 7C(b) presents a simplified schematic of this configuration, along with several views of the functionalized SuC-PCF. The probe successfully detected the ovarian cancer marker haptoglobin (Hp) in ovarian cyst fluid and demonstrated potential in differentiating between various stages of ovarian cancer, showing a strong correlation with the results of clinical detection methods. This work layed a solid foundation for the development of a highly sensitive SERS-activated photofluidic biopsy needle.

Furthermore, Eravuchira et al. [71] integrated HC-PCF with SERS technology to create a highly sensitive tool for detecting amyloid β-peptide (Aβ42), a key biomarker of Alzheimer’s disease. In this study, an optical transmission platform based on HC-PCF was established to conduct Raman spectroscopy detection experiments. The experimental setup comprises two main components: a Labram HR-type confocal Raman spectrometer, manufactured by a Japanese company, and a custom-designed and fabricated optical fiber fixation device for aligning and securing the HC-PCF, as shown in Figure 7D. By leveraging the SERS technique to amplify the Raman signal, the researchers achieved substantial signal enhancement with only 30 μL of sample—addressing the small-volume requirement for CSF samples noted in in vivo SERS reviews [9]. This advancement paves the way for the development of a sensitive, label-free detection tool, which holds great promise for the early diagnosis of Alzheimer’s disease in the future.

#### 4.2.2. Cellular Correlation

In the realm of analytical science, SERS techniques have emerged as a powerful tool with remarkable capabilities at the cellular level. Over the years, these techniques have witnessed significant advancements, enabling diverse applications such as tumor cell detection and the identification of pathogenic microorganisms. The scope of these applications is extensive, ranging from quantitative monitoring of carboxylesterase-1 in hepatocellular carcinoma cells to precise identification of circulating tumor cells. Additionally, SERS techniques facilitate in situ detection of heat shock protein mRNA in living cells and single-cell analysis of drug resistance in Mycobacterium tuberculosis [72,73,74,75]. However, cellular samples present several challenges, including complex compositions, low target concentrations, and susceptibility to matrix interference. These factors have long posed a significant hurdle to achieving highly sensitive and specific detection in the field of analytical science.

To address these challenges, a variety of detection methods have been developed for cellular analysis. These include fluorescence imaging [76], flow cytometry [77] and mass spectrometry [78], Despite their widespread use, these methods still face limitations when it comes to achieving single-cell resolution and multiplex detection capabilities. In recent years, the integration of SERS technology with optical fibers has emerged as a novel and promising solution. This integration involves the development of innovative fiber-optic SERS probes. These probes have significantly enhanced detection sensitivity and specificity, thereby providing a powerful analytical tool for cellular research.

**Table 3:** Applications of PCF-SERS sensors in biomedicine.

Analytes	Type of Optical Fiber	SERS Active Nanomaterials	Limit of Detection	Ref.
epidermal growth factor receptors in a lysate solution from human epithelial carcinoma cells	Hollow core PCF	AuNP-MGITC-antibody	100 pg/mL	[68]
hepatocellular carcinoma biomarkers-alpha fetoprotein and alpha-1-antitrypsin	Hollow core PCF	AuNP-Cy5 or AuNP-MGTIC antibody	N.A.	[69]
haptoglobin, a biomarker for ovarian cancer	Suspended core PCF	AuNPs	N.A.	[70]
amyloid β (1–42) peptide (Aβ42), a major AD biomarker	Hollow core PCF	AuBPs	40 μg/mL	[71]
acute myeloid leukemia cells	Hollow core PCF	AgNPs	300 cells/mL	[79]
lipid-peroxidation-derived protein modifications in cells	Side channel PCF	AuNPs	0.7 µg/mL	[80]
sialic acid on single cell	Side channel PCF	AuNPs	2.5 fM	[40]
amino acid neurotransmitters	Hollow core PCF	AuNPs	10^−4^ M	[81]

N.A.: Not Applicable (not relevant to the corresponding item).

**Figure 8:**
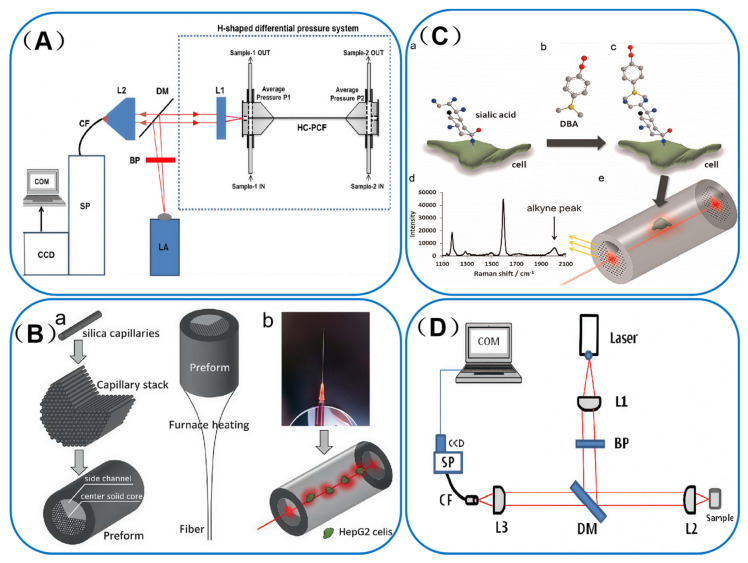
(**A**) Schematic of the setup. LA: Laser source with collimating lens; BP: Band pass filter; DM: Dichroic Mirror; L1: Microscope objective lens for light coupling; H-shaped differential pressure system with hollow core photonic crystal fiber; L2: Microscope objective lens for backward light collection; CF: Collection fiber; SP: spectrograph; CCD: CCD camera; COM: Computer. Adapted with permission from ref. [79]. (**B**) Scheme of SC-PCF sensor. Adapted with permission from ref. [80]. (**C**) Illustration of the detection method. Adapted with permission from ref. [40]. (**D**) Schematic of the set-up. L1: Collimating lens (shapes 785 nm excitation laser to initiate SERS); BP: Band pass filter (isolates laser wavelength); DM: Dichroic Mirror (separates excitation light and Raman signals); L2: Microscope objective (focuses laser onto HC-PCF sample to induce SERS); L3: Microscope objective (collects backward SERS signals); CF: Collection fiber (transmits SERS signals to spectrograph); SP: Spectrograph (disperses SERS spectra); COM: Computer (data acquisition/analysis). Adapted with permission from ref. [81].

One notable study in this field was conducted by Altaf Khetani et al. [79] who focused on integrating HC-PCF with SERS technology to develop a highly sensitive detection platform for leukemia cells. By incorporating silver nanoparticles into the HC-PCF, the researchers achieved a significant enhancement of the Raman signal. This advancement enabled efficient and sensitive detection of leukemia cells. The detailed design of the HC-PCF sensor is illustrated in Figure 8A. This innovative approach not only improved the sensitivity of the assay but also minimized sample consumption. The results demonstrated that the platform could effectively differentiate between various physiological states of leukemia cells, such as apoptosis, survival, and necrosis. Moreover, it could accurately detect leukemia cells at concentrations as low as 300 cells/mL. This achievement paves the way for sensitive, label-free detection of leukemia cells and is expected to play a crucial role in future clinical diagnosis and monitoring.

In a similar vein, Gong et al. [80] designed a novel type of PCF known as SC-PCF. This unique fiber structure allows liquid samples to be pumped through the side channels, while light is confined and transmitted through a solid core at the center. In this study, the side-channel PCF was innovatively employed as a SERS substrate, enabling the successful and precise monitoring of intracellular modifications of lipid peroxidation-derived proteins. Figure 8B(a) presents a schematic diagram of the preparation process for the homemade side-channel PCF, while Figure 8B(b) details the fabrication process of the sensor for SERS detection. Through SERS measurements and mapping, the researchers were able to successfully characterize the lipid antioxidant LAA (lipid peroxidation-derived product) and compare the SERS detection efficacy with old and new optical fiber designs. They found that the newly designed fiber, with its smaller pitch and core diameter, could produce stronger extinction light, thereby enhancing the SERS detection intensity. In addition, the study explored the cholesterol-induced lipid peroxidation process and observed the intracellular distribution of LAA. These findings demonstrate that the proposed fiber-optic SERS technique has the potential to detect lipid peroxidation at the cellular level, offering new perspectives and powerful tools for relevant cellular studies and applications.

Tianxun Gong et al. [40] developed an ultrasensitive SERS-based sensing platform for detecting salivary acid levels on cell surfaces, achieving a remarkable sensitivity of up to 2 femtomoles. The platform utilizes an interference-free Raman tag, DBA. As illustrated in Figure 8C(b), DBA comprises a salivary acid trapping group (phenyl-boronyl group) and an alkyne functional group. This tag selectively binds to the cell membranes of salivary acid-binding sites, as shown in Figure 8C(a,c). It leverages interference-free alkyne Raman peaks for detection, as depicted in Figure 8C(d). This innovative approach enables highly specific and sensitive SERS detection of salivary acids. The platform holds great promise as a clinical diagnostic tool for real-time analysis of salivary acid-related tumors.

Furthermore, Vidhu et al. [81] used SERS for detecting neurotransmitters such as glutamate (GLU) and gamma-aminobutyric acid (GABA). These neurotransmitters mediate excitatory and inhibitory neurotransmission in the brain, respectively, and play crucial roles in neuroendocrine control. Disruptions in their synthesis have been associated with neurological disorders such as epilepsy. The SERS protocol can detect low concentrations of GLU (10^−7^ M) and GABA (10^−4^ M). This high sensitivity is expected to complement existing techniques, such as high-performance liquid chromatography (HPLC) and mass spectrometry (MS). The SERS method, with its sample preparation simplicity, molecular specificity, and high sensitivity, can be effectively used for characterizing samples such as brain extracts, cerebrospinal fluid, and saliva. Figure 8D shows their SERS-specific experimental setup, designed for the SERS-based neurotransmitter detection workflow mentioned earlier. This setup integrates HC-PCF, using its photonic bandgap to strengthen light-sample interaction. It also includes the 785 nm excitation laser, critical for activating SERS. This design enables sensitive detection of GLU and GABA in microliter aqueous solutions, matching the trace sample needs of clinical specimens noted before.

#### 4.2.3. Antibiotics

Antibiotics are of paramount importance in medical treatment, especially in combating severe infections. They serve as vital tools for controlling pathogen growth, curbing disease progression, safeguarding vital organ functions, and ultimately rescuing patient’s lives. Nevertheless, the therapeutic efficacy of antibiotics is contingent upon sustaining appropriate concentrations within the patient’s body. In critically ill patients, marked changes in physiological conditions, such as drug distribution, plasma protein binding, and hepatic and renal metabolism, can induce significant fluctuations in antibiotic plasma levels. These pharmacokinetic alterations escalate therapeutic uncertainty and the risk of drug toxicity, underscoring the necessity for precise monitoring and customized treatment strategies [82,83].

At present, the determination of antibiotic levels in body fluids predominantly relies on techniques based on chromatography [84] and electrophoresis [85]. These methods are not only time-consuming but also demand intricate sample preparation procedures. Coupled with the time required for sample transportation to central laboratories, the entire process often results in a delay of several hours or even days before results become available. Such a delay precludes the immediate adjustment of antibiotic dosages. Consequently, the precise regulation of antibiotic dosage and the implementation of individualized treatment plans have become imperative to enhance therapeutic efficacy and minimize adverse effects. The medical community is actively engaged in the development of efficient and sensitive technologies for detecting antibiotic concentrations. These advancements are expected to facilitate immediate and precise monitoring, providing a robust foundation for clinical decision-making and timely adjustments of treatment strategies. They hold the promise of ensuring the safe and effective utilization of antibiotics, which is a critical priority in the current medical landscape of disease management [86,87].

Raman spectroscopy, a non-invasive analytical technique, holds great potential for highly chemo selective analysis of biomolecules and pharmaceutical compounds. Yet, its application in analyzing low-concentration solutions is often limited by the inherently weak Raman scattering signal. This limitation necessitates signal enhancement. HC-PCF has emerged as a promising solution to this challenge. With its hollow core and surrounding periodic array of air holes, HC-PCF features a cladding structure that creates an optical band gap, allowing it to guide a specific spectral region. In recent years, HC-PCF has shown significant progress in enhancing Raman sensing. The simplest approach involves filling the entire HC-PCF structure with liquid. However, when the air holes are filled with liquid, the band gap shifts to shorter wavelengths due to changes in the refractive index contrast. This shift effectively directs the optical signal, thereby enhancing the Raman signal through fiber-optic mechanisms.

The Leibniz Institute for Photonic Technologies in Jena, Germany [88], has delved into the properties of HC-PCF and evaluated their potential in Raman spectroscopy, particularly for monitoring antibiotics. This research has enabled the sensitive detection of moxifloxacin, a clinically important antibiotic drug. By transmitting and collecting backscattered Raman signals via optical fibers, the researchers successfully enhanced the Raman signals. This breakthrough has enabled the detection of moxifloxacin at extremely low concentrations. Compared to conventional chromatography and electrophoresis, this technique is significantly faster and requires substantially less sample material.

Table 3 and the accompanying biomedical case studies confirm that HC-PCF integrated with AuNPs is the prevailing architecture for SERS-based biomedical sensing. Its dominance rests on structural–material features that match the constraints of biological specimens, while built-in trade-offs show that “reliability” is prioritised over “ultra-sensitivity” in clinical scenarios.

These same features set the AEF of HC-PCF apart from conventional Au/Ag substrates. Traditional biomedical Au/Ag surfaces (e.g., antibody-functionalised Ag films) typically offer an AEF of 10^6^–10^7^, limited by non-specific binding and short light–analyte interaction times. Even in the presence of biological matrix interference (cell lysates, cyst fluid, etc.), HC-PCF-SERS retains a one-order-of-magnitude advantage, delivering ~10^8^ for lipid-peroxidation-derived proteins and single-cell sialic acid outperforming standard Au-NP bioprobes. Although the HC-PCF AEF in biomedicine is lower than in chemical sensing because the matrix weakens electromagnetic hot-spots, it still exceeds that of conventional substrates, providing a balanced compromise between sensitivity and reliability.

HC-PCF is widely adopted for biomarker detection (EGFR, Aβ42, Table 3) because the hollow core allows direct loading of minute biological volumes (e.g., cerebrospinal fluid for Aβ42). This geometry circumvents two key shortcomings of SC-PCF in biomedical work: analyte loss during diffusion into cladding pores and non-specific interactions with the solid silica core that raise background signals. The only minor drawback of HC-PCF is its sensitivity to the high refractive index of biological fluids such as blood plasma, which can slightly elevate LODs relative to SC-PCF under ideal conditions.

AuNPs are the default choice in bio-analysis: every biomedical target in Table 3—haptoglobin, sialic acid, leukaemia cells—rides on gold, while silver is virtually absent. Their inert surface survives oxidation by lysates, cyst fluid or serum, so the SERS tag lasts long enough to be read. The price is a lower LSPR multiplier than Ag, hence biomedical LODs sit a decade above those recorded for clean chemical matrices; the sacrifice buys reproducible signal in the body’s chemical chaos.

For single-cell applications, specialised fiber geometries underscore the primacy of “sample compatibility” over absolute sensitivity. Side-channel SC-PCF enables real-time tracking of intracellular lipid-peroxidation-derived protein modifications by delivering samples through side channels, eliminating the cell damage that would result from direct core filling-a prerequisite for live-cell assays. AgNP-doped HC-PCF proves the exception: it trades a picomolar LOD for a cytocompatible, low-cost leukaemia-cell assay, showing that biomedical fibre design answers to budget and cell viability, not to the ultra-trace chase that rules chemical sensing. Table 3 summarizes the reported PCF-SRES based biomedicine.

### 4.3. Detection of Explosives

SERS has revolutionized the field of analytical science by surmounting the conventional Raman scattering’s weak signal hurdle, attributing to its distinctive enhancement mechanism. This breakthrough has thrust it into the limelight as a pivotal technology, with extensive applications spanning drug and explosives detection, thereby playing a critical role in upholding public health and safety.

In recent times, the escalating frequency of bombing attacks has posed an unprecedented challenge to public security. Consequently, explosives detection has emerged as the epicenter of public security research endeavors. The ramifications of achieving rapid and precise detection of explosives are monumental, given the potential to thwart threats and safeguard lives and infrastructure. Against this backdrop, the advent of SERS technology has heralded a new era in explosives detection. Its unique strengths, particularly in discerning the molecular fingerprints of explosives, enable the detection of ultra-trace quantities and facilitate real-time identification. These attributes have rendered it an indispensable tool, affording highly efficient technical support across diverse scenarios such as security screening and forensic investigation [89,90].

SERS has cemented its position as one of the most rapidly advancing ultra-sensitive vibrational fingerprinting techniques in recent years. This assertion is bolstered by its proven efficacy in detecting a broad spectrum of molecules and biomolecules. Notably, its exceptional performance in detecting minute traces of TNT (trinitrotoluene) and DN (dinitrotoluene) has been demonstrated through extensive research and practical applications [91]. For instance, Zhang et al. devised an innovative strategy to construct the charge-transfer TNT-PABT complex on top-enclosed flexible silver nanotube arrays. This ingenious approach has functioned as a formidable “signal booster” for Raman-inactive TNT, culminating in a remarkable ultra-low detection limit of 1.5 × 10^−17^ M [92]. While SERS enables highly sensitive detection of trace explosives like TNT, most processes remain manual-inefficient and exposing operators to toxic risks. To address this, Liu et al. integrated SERS with digital microfluidics (SERS-DMF) for automated, high-throughput, sensitive detection. A DMF chip (40 driving/8 storage electrodes) facilitated sample processing, featuring automated droplet handling and salt-optimized silver nanoparticle (Ag NP) aggregation to generate SERS “hotspots”. The platform achieved detection limits of 10^−7^ M (TNT) and 10^−8^ M (NTO), with superior reproducibility and efficiency compared to manual methods, enhancing SERS’ practical value in public security [93].

**Figure 9:**
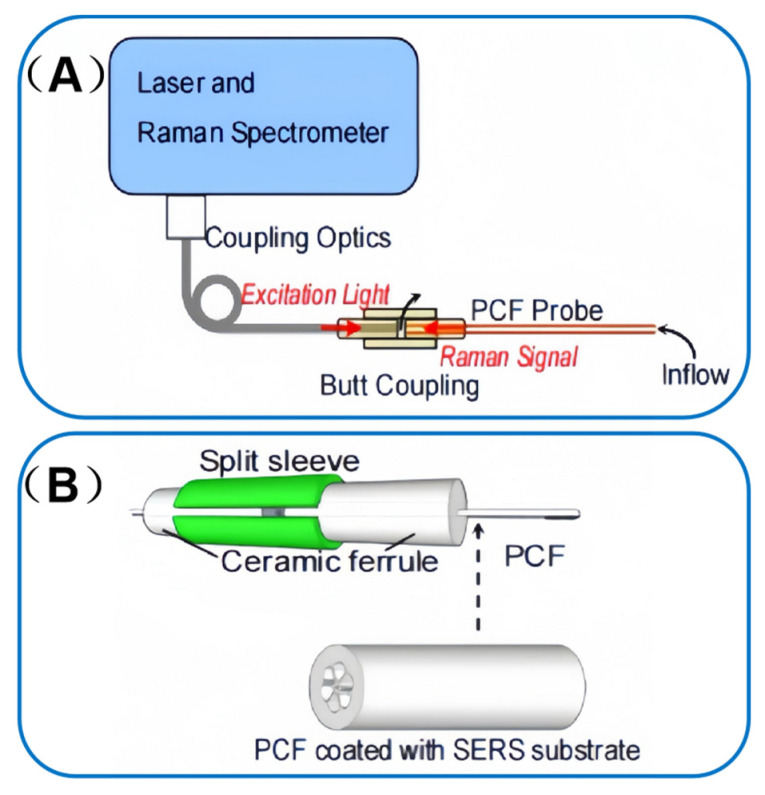
(**A**) Schematic diagram of Raman spectroscopy combined with PCF-SERS probe to detect the gaseous analyte. (**B**) Schematics of butt coupling and the grapefruit PCF coated with SERS substrate on its inner wall. Adapted with permission from ref. [48].

Beyond these advancements in automation, the integration of SERS with PCF has further expanded the frontiers of explosive molecule detection. Chuanyi Tao et al. [48] pioneered the use of gold nanoparticles as a PCF-SERS substrate for probing TNT vapors. A standard optical fiber is employed to couple the laser to the Raman spectrometer, facilitating the delivery of laser light and the collection of Raman signals from the PCF-SERS probe, as illustrated in the schematic diagram of Figure 9A. The fabrication process of the SERS substrate, as shown in Figure 9B, involves polymerization-mediated self-assembly to form a monolayer of gold nanoparticles within the PCF air channel. This meticulously engineered substrate boasts several salient advantages. Its high SERS enhancement capability serves to amplify the detection signal, ensuring that even minute traces of explosives can be discerned. The substrate’s high affinity for explosive vapors enables precise and selective target capture, minimizing false positives and enhancing detection accuracy. Furthermore, its rapid response to adsorption/desorption processes ensures timely feedback, which is crucial in time-sensitive detection scenarios.

The PCF-SERS probe has exhibited exceptional sensitivity, surpassing that of conventional fiber-optic-less probes employed for direct focused laser detection. This enhanced sensitivity can be attributed to the unique optical confinement within the air channel, where the excitation light, Raman scattering light, and analyte are spatially confined. This confinement substantially increases the effective interaction volume between the light and the analyte molecules, thereby amplifying the Raman signal.

Notably, the light confinement advantage of PCF’s air channel—key to amplifying Raman signals for explosive detection—can be further enhanced via tailored hollow-core subtypes. Nie et al. [94] specified that for gas-phase explosive targets, hollow-core photonic bandgap fibers (HC-PBFs) boost signal collection with a large numerical aperture, while hollow-core anti-resonant fibers (HC-ARFs) suppress silica background via single-mode transmission. Paired with low near-infrared loss, HC-ARFs achieve low limits of detection for dilute explosive vapors; micron-scale pinhole spatial filtering further mitigates cladding silica interference, elevating target vapor signal-to-noise ratio. This work refines PCF subtype selection for explosive gas-phase sensing and offers targeted technical optimization for air channel-based signal enhancement, strengthening the on-site practicality of PCF-SERS probes.

The outcomes of this research carry profound implications. They are anticipated to catalyze the development of rapid and highly sensitive fiber-optic SERS devices. Such advancements hold the potential to transcend the limitations of traditional laboratory-based detection methods. By enabling on-site identification and monitoring of explosive molecules, this technology could revolutionize field-based detection protocols. Ultimately, it is envisioned that this innovation will drive further advancements in the sensor field and furnish robust technical support for explosive detection, thereby enhancing the overall landscape of public safety measures.

## 5. Conclusions and Future Perspectives

Over the past decade, SERS has matured into an ultra-sensitive, fast and low-cost analytical tool widely adopted for environmental, food and biomedical assays. This review highlights the accelerating progress of PCF-based SERS sensors that combine extreme sensitivity (ppt level), small flexible footprints and easy integration into microfluidics or wearables. Yet variability in nanoparticle synthesis, surface functionalisation and matrix effects still undermines reproducibility, while fluorescence, non-specific binding and the absence of calibration standards complicate quantification, especially in complex biological samples.

This review examines how SERS merges with HC-PCF and SC-PCF. It critically evaluates recent solid-core, hollow-core and side-channel SERS-PCF sensors and their growing roles in chemical detection, biomedicine and explosive identification. Embedding SERS-active zones inside micro-structured HC-PCF or SC-PCF removes long-standing limits of conventional SERS: weak light confinement, bulky optics and poor in situ compatibility.

Remaining problems stem from fiber architecture, not universal SERS issues. In HC-PCF, plasmonic films on tortuous pores are uneven and degrade reproducibility. SC-PCF loses excitation power at the fiber-source interface. Across all designs, the absence of on-fiber sample handling lets complex matrices such as serum or post-blast residues create interference and non-specific binding that obscure trace analytes.

Coming research will target three pillars: Precision structures, AI-driven multifunctional systems, Scalable manufacturing and field deployment.

1. Precision structures

Atomic Layer Deposition delivers angstrom-scale Au or Ag films on HC-PCF inner walls and SC-PCF multi-core claddings. Anti-fouling PEG or zwitterionic brushes suppress non-specific binding in blood or environmental extracts.

2. AI-driven multifunctional systems

CNN-LSTM hybrids trained on noisy, overlapping spectra from bodily fluids or explosive mixtures yield one-shot analyte IDs. Three-dimensional direct-laser-written microfluidic networks inscribed in the HC-PCF or SC-PCF cladding enable autonomous sampling, filtering and reagent mixing.

3. Scalable manufacturing and field deployment

Roll-to-roll thermal drawing of polymer HC-PCF and SC-PCF produces kilometer lengths compatible with standard fiber cabling. Pocket-sized 785 nm NIR lasers and CMOS spectrometers clipped to the fiber pigtail allow on-site environmental surveillance or security checkpoints. Bioresorbable plasmonic coatings on silica HC-PCF or SC-PCF permit transient implantation for intraoperative tumor-margin mapping with minimal inflammatory response.

**Figure 10:**
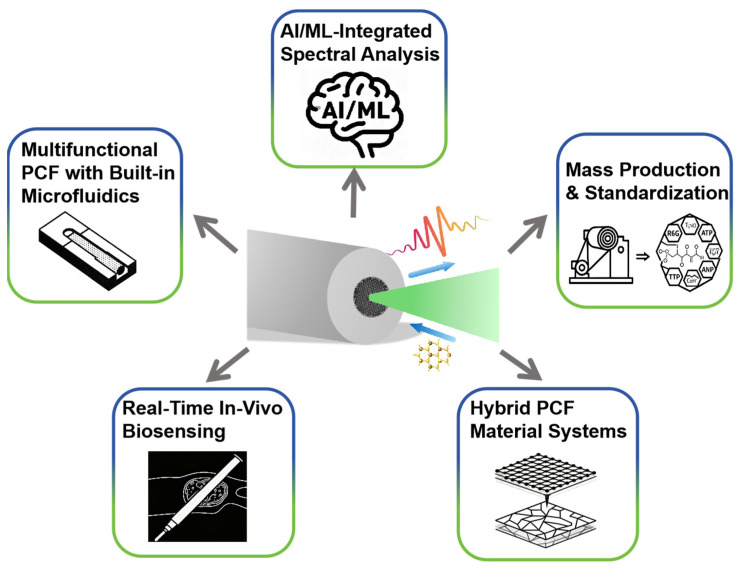
Overview of PCF-SERS sensors Future Development Directions.

By uniting SERS molecular specificity with HC-PCF and SC-PCF mechanical flexibility and miniaturization, these SERS-PCF-tailored actions can shift the technology from bench-top curiosity to field-deployable tools for precision medicine, food safety and environmental protection. Figure 10 provides an overview of the future development directions for PCF-SERS sensors.

## Figures and Tables

**Table 1:** Comparison of the advantages and limitations between traditional SERS sensors and PCF-SERS sensors.

Category	Advantages	Limitations
traditional SERS sensors	Rapid detection Reproducible	Lack of flexibility and compactness rigid and bulky design Limited application
PCF-SERS sensors	Flexible design Increase the volume of the active sensing area Anti-interference Lower Raman background High spectral certainty	The level of technological maturity needs to be improved Multi-parameter cross-sensitivity

**Table 2:** Applications of PCF-SERS sensors in chemical substances.

Analytes	Type of Optical Fiber	SERS Active Nanomaterials	Limit of Detection	Analytical Enhancement Factor	Ref.
RhB	Solid core PCF	AuNPs	10^−7^ M	~10^6^	[45]
R6G	Hollow core PCF	AgNPs	10^−10^ M	~10^8^	[54]
R6G	Solid core PCF	AgNPs	10^−10^ M	~10^7^	[55]
R6G	Side channel PCF	AuNPs	5 × 10^−14^ M	~10^9^	[56]
ATP	Tapered Suspended Core PCF	AuNPs	1 × 10^−7^ M	~10^7^	[42]
CV	Suspended core PCF	AgNPs	10^−12^ M	~10^11^	[57]
